# Parasitemia and Associated Immune Response in Pregnant and Non-Pregnant Beef Cows Naturally Infected With *Neospora caninum*

**DOI:** 10.3389/fvets.2022.905271

**Published:** 2022-06-14

**Authors:** Ignacio Gual, Lucía María Campero, Yanina Paola Hecker, Javier Regidor-Cerrillo, María Rosa Leunda, Anselmo Carlos Odeón, Carlos Manuel Campero, Susana Torioni de Echaide, Ignacio Eduardo Echaide, Silvia Marcela Estein, Luis Miguel Ortega-Mora, Dadín Prando Moore

**Affiliations:** ^1^Facultad de Ciencias Agrarias, Universidad Nacional de Mar del Plata, Balcarce, Argentina; ^2^Instituto de Innovación para la Producción Agropecuaria y el Desarrollo Sostenible (IPADS Balcarce), Instituto Nacional de Tecnología Agropecuaria Estación Experimental Agropecuaria Balcarce (CONICET-INTA), Balcarce, Argentina; ^3^Grupo SALUVET, Departamento de Sanidad Animal, Facultad de Ciencias Veterinarias, Universidad Complutense de Madrid, Madrid, Spain; ^4^Instituto Nacional de Tecnología Agropecuaria Estación Experimental Agropecuaria Balcarce, Balcarce, Argentina; ^5^Instituto Nacional de Tecnología Agropecuaria Estación Experimental Agropecuaria Rafaela, Rafaela, Argentina; ^6^Centro de Investigación Veterinaria Tandil, Facultad de Ciencias Veterinarias, Universidad Nacional del Centro de la Provincia de Buenos Aires (CONICET-UNCPBA), Tandil, Argentina

**Keywords:** pathophysiology, *Neospora caninum*, cattle, immune response, parasitemia

## Abstract

The aim of this longitudinal study was to characterize the parasitemia of *Neospora caninum* and the associated immunological parameters in naturally infected beef cows for 10 months. The following groups were established: *Neospora caninum* seropositive pregnant cows (+Preg, *n* = 7), seropositive non-pregnant cows (+Npreg, *n* = 7), seronegative pregnant cows (−Preg, *n* = 4), and seronegative non-pregnant cows (−Npreg, *n* = 4). Several samples were obtained for absolute and relative leukocyte counting, cytokines IL-10, IL-12, α-TNF, and γ-IFN quantification, specific IgG, IgG1, and IgG2 and avidity and *N. caninum* DNA molecular detection and quantification. The +Preg group had a higher frequency and concentration of *N. caninum* DNA in PBMC in the last third of pregnancy compared to +Npreg (*p* <0.05), with 22 and 8% of detection, respectively. Parasitemia correlated positively with IgG titers and negatively with IgG1/IgG2 ratio (*p* <0.05). On day 222 of the assay, the +Preg group had the lowest total leukocyte counting (*p* <0.05). The +Preg group had a higher concentration of IgG and higher avidity in the last third of gestation compared to +Npreg (*p* <0.05). Avidity correlated with total IgG and IgG2 (*p* <0.05). All +Preg cows gave birth to clinically healthy but seropositive calves before colostrum intake, therefore, the congenital transmission was 100% efficient. Only a complete *N. caninum* genotype from a placenta and a partial genotype from cow #3 of the group +Preg were achieved by multilocus microsatellite analysis. Overall, *N. caninum* parasitemia is frequent in seropositive beef cows during the last third of gestation. This correlates with higher antibody levels and a decrease in total leukocyte counting. The precise timing of the parasitemia may be used for diagnosis purposes and/or for design strategies to avoid vertical transmission. Further studies are needed to identify the immune molecular mechanisms that favor parasitemia during gestation in chronically infected cattle.

## Introduction

*Neospora caninum* is an intracellular parasite responsible for abortion in cattle. In chronically infected cattle, the parasite lives within tissue cysts. Immunological and hormonal changes that take place at the gestation of cows favor the parasite recrudescence and consequently transmission to their offspring. This efficient transmission contributes to the maintenance of neosporosis in herds and is associated with the endemic pattern of abortion (1). To date, control measures are limited since there is no vaccine or pharmacological treatment available to prevent abortion and transmission. Therefore, the prophylaxis is based on management measures aiming to interrupt the life cycle of *N. caninum* (2).

Many advances in the knowledge of bovine neosporosis have been achieved since its first diagnosis in 1989 (3), however, there are still some aspects that require further investigation. These are related to parasite reactivation in tissue cysts and the parasitological and immunological mechanisms associated with parasitemia during pregnancy. An increase in antibody level at mid-gestation has been described as indicative of recrudescence (4) but no study corroborates it yet. Although the role of antibodies is still unclear and there is scarce information on *N. caninum* parasitemia, it has been proposed that antibodies would help to neutralize the parasite and avoid cellular invasion at extracellular stages (4).

During parasitemia, *N. caninum* travels intracellularly within peripheral mononuclear blood cells (PBMC) (5) but there are also some reports on *N. caninum* DNA in sera (6, 7). Even though most worldwide *N. caninum* isolations were obtained from brain samples, Bień et al. (8) and Hao et al. (9) could isolate from PBMC of bison and cattle, respectively. The presence of *N. caninum* DNA in blood from pregnant (5, 6, 10–13) and non-pregnant (14–16) naturally infected cattle has been partially studied. Most of the studies on *N. caninum* parasitemia focus only on a brief period or a single moment of gestation, thus characterizing a fragmented and biased scenario of parasitemia. Hence, parasitemia evaluation throughout the entire gestation period has not yet been studied. Studies on parasitemia (duration, timing, and quantification) and the immunological response would help to better understand the physiopathology involved in bovine neosporosis. The main objective of this study was to characterize *N. caninum* parasitemia and the associated immunological response in naturally infected pregnant and non-pregnant beef cows throughout the entire gestation/10-month period.

## Materials and Methods

### Animals and Experimental Groups

A total of twenty-two beef breed cows raised at INTA EEA Balcarce (37°46′34″S; 58°13′25″O) were grouped according to the serological results for *N. caninum*-specific antibody detection obtained from indirect fluorescence antibody test (IFAT) (cutoff titer ≥1:200) (17) and a commercial indirect ELISA (iELISA) (CIVTEST® BOVIS *Neospora*, HIPRA, Girona, Spain). The cows used in this study belonged to 3 herds. The cow's body condition was evaluated on a scale from 1- 5 throughout the study (18).

The estrus was synchronized by two applications of 2 ml of F2α D-cloprostenol (0.075 mg/ml, Baker®, Tecnofarm) with an interval of 11 days. Four Aberdeen Angus bulls (seronegative to *N. caninum*) were used for natural mating for 3 days released at 48 h from the second application of F2α D-cloprostenol. The gestation diagnosis was performed by ultrasonography (Honda HS-101V, Japan) 40 days after mating. Fetal viability was monthly monitored by transrectal ultrasonography during the first trimester of gestation and by transrectal palpation onward until delivery estimated time.

The following experimental groups were set up based on the serological condition regards to *N. caninum* (seropositive/seronegative) and the physiological stage (pregnant/non-pregnant):

a) Seropositive pregnant cows (+Preg, *n* = 7).
b) Seropositive non-pregnant cows (+Npreg, *n* = 7).
c) Seronegative pregnant cows (-Preg, *n* = 4).
d) Seronegative non-pregnant cows (−Npreg, *n* = 4).

The animals were clinically and sexually suitable for reproduction, seronegative to *Brucella abortus* and *Toxoplasma gondii*, and free of tuberculosis, trichomoniasis, and campylobacteriosis. The animals received the obligatory vaccines for brucellosis, carbuncle, and foot and mouth disease according to the National Service for Food Health and Quality (SENASA) sanitary calendar regulation. Furthermore, serology to Bovine Viral Diarrhea (BVD), Infectious Bovine Rhinotracheitis virus (IBR), and leptospirosis (*Leptospira interrogans*) was performed every 3 months and no seroconversion was recorded.

The animals used in this study were handled in strict accordance with the good animal practice and the conditions were approved by the Animal Ethics Committee at INTA Balcarce (CICUAE#009/2015).

### Experimental Design and Sampling

**Figure 1:**
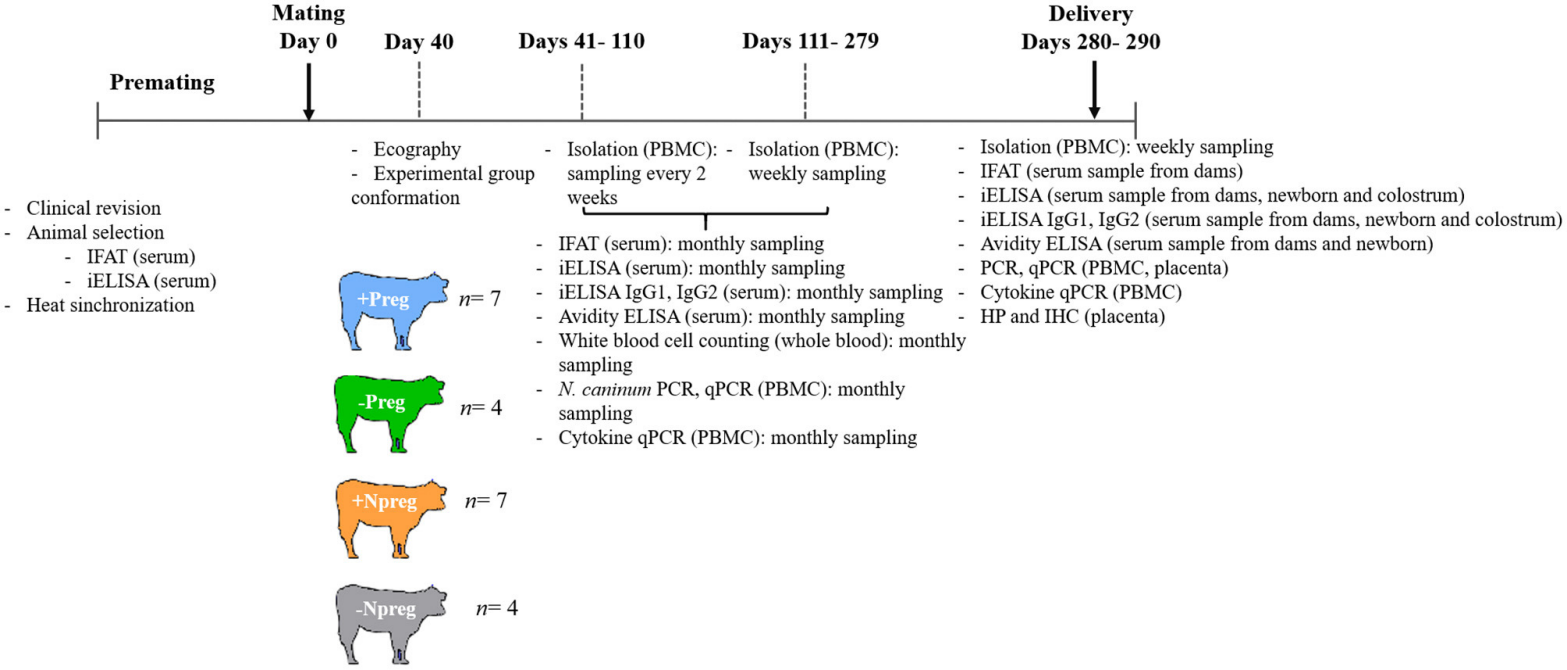
Experimental design, sample schedule, and procedures carried out.

A 10-month longitudinal study was performed considering day 0 as the first day of mating. Figure 1 summarizes the experimental design, sample schedule, and procedures carried out throughout the study. Blood samples with (30 ml) and without (10 ml) EDTA (Wiener Laboratorios S.A.I.C., Rosario, Argentina) were collected by jugular vein puncture. Anticoagulated blood samples were immediately processed by a density gradient (Histopaque, Sigma, United States). Peripheral blood mononuclear cells (PBMC) were counted in the Neubauer chamber with trypan blue (1:2), fractionated for different analyses, and a subsample stored at −80°C for further analyses (see below). Blood samples without EDTA were centrifuged at 1,600 × g for 10 min and serum was stored at −20°C.

### Specific Humoral Immune Response: IFAT, IgG, Avidity, and IgG1 and IgG2 Subclasses

Serological assays were performed on serum samples collected on days 0, 40, 65, 95, 125, 155, 188, 222, 246, and 280 of the study. The IFAT was performed according to Campero et al. (17) and a cutoff titer ≥1:200 was used. The commercial *N. caninum* iELISA (CIVTEST® BOVIS *Neospora*, HIPRA, Girona, Spain) was used for specific IgG detection following the manufacturer's instructions. For the interpretation of the results the relative index percent (RIPC) was calculated according to the following formula: OD_405_ sample—Mean OD_405_ Negative Control/Mean OD_405_ Positive Control—Mean OD_405_ Negative Control x 100. The cutoff was expressed as RIPC >10 and RIPC >4.74 for serum and colostrum samples, respectively. Furthermore, avidity was evaluated by the commercial IgG avidity ELISA (CIVTEST® BOVIS *Neospora*, Avidity Supplement), and results were expressed as Rz value, according to the manufacturer. Rz value ≤ 1 indicates high avidity, 1-2 intermediate and ≥2 low avidity.

*Neospora caninum* subclasses IgG1 and IgG2 were determined by iELISA as previously described by Moore et al. (19). Anti-bovine IgG1 or IgG2 monoclonal antibodies (mAbs) were used 1:100 (Serotec^TM^, Oxford, United Kingdom). Data were expressed as the ratio of OD values for IgG1/IgG2.

### Non-Specific Immune Response

#### Leukocyte Counting

White blood cell counting was performed on blood samples obtained on days 65, 95, 125, 155, 188, 222, and 246. Total leukocyte counts were manually assessed in the Neubauer chamber. The blood sample was diluted 1:20 using Turk's fluid, composed of 1% acetic acid and 0.1% gentian violet. The total leukocyte number was multiplied by 50 to obtain the total number of leukocytes per ml of blood (20).

Differential counts for lymphocytes, neutrophils, eosinophils, and monocytes were obtained from blood smears stained with May Grunwald-Giemsa (Merck, United States) under the light microscope. A total of one hundred leukocytes per smear were observed every 10,000 leukocytes/mm3 (20).

#### Cytokine mRNA Expression

Cytokine quantification of interferon-γ (IFN-γ), interleukin-12, tumor necrosis factor-α (TNF-α), interleukin-10 (IL- 10) from PBMC samples obtained at days 65, 125, 155, 188, and 222 was performed. Total RNA was extracted from 2.5 ×10^6^ PBMC preserved in 375 μl of Trizol® (Ambion, Life Technologies, California, United States), following the manufacturer's instructions. RNA was digested with DNase I Amplification Grade (Invitrogen, Carlsbad, CA, United States) for 30 min at 37°C to remove any contaminating genomic DNA (gDNA). The quality and quantity of the resulting RNA were determined using an Epoch Microplate Spectrophotometer (BioTeK, Winooski, VT, United States). All RNA samples were stored at −80°C until cDNA synthesis was performed. Synthesis and amplification of cDNA were performed by PCR according to Hecker et al. (21). Real-time PCR was performed using primers for bovine interferon-γ (IFN-γ), interleukin-12 p40, tumor necrosis factor-α (TNF-α), interleukin-10 (IL- 10), and the housekeeping gene β-actin on cDNA samples from PBMC, as previously reported (22). Relative quantification of cytokine mRNA expression levels was carried out using the 2–ΔΔCT method (23).

### *Neospora caninum* Molecular Detection

Detection of *N. caninum* by PCR in PBMC was performed on days 40, 65, 95, 125, 155, 188, 222, 246, and 280 of the study. DNA was extracted from 5 ×10^6^ PBMC in 100 μl of PBS, and 25 mg of placental tissue using a commercial kit (WizardR Genomic DNA Purification Kit, Promega, United States) according to the manufacturer's protocol. A conventional PCR with standard reagents (Productos Bio-Lógicos, Argentina) was performed using the pair of primers Np6+/Np21+ (Invitrogen, United States) amplifying a 328 bp fragment (24). PCR reaction mixture (25 μl) contained 2.5 μl of 10X DNA polymerase reaction buffer; 19.675 μl of water; 0.625 μl of 10 mM dNTPs; 0.125 μl of 100 μM for each primer; 0.75 μl of 50 mM MgCl_2_; 0.2 μl of 5 u/μl DNA polymerase enzyme and 1 μl of sample DNA. DNA from *N. caninum* NC-1 isolate was used as positive control and DNA from VERO cells as negative control. PCR was performed in a thermocycler (Applied Biosystems, United States) according to the following conditions: 94°C for 5 min; 35 cycles of 94°C for 30 s; 56°C for 30 s, and 72°C for 60 s. PCR results were analyzed by 2% agarose gel electrophoresis stained with SYBR safe (Invitrogen, United States) and compared to a standard molecular weight marker of 100 bp ladder (Productos Bio-Lógicos, Argentina).

Positive samples to conventional PCR were quantified by real-time PCR (qPCR), using the pair of primers that amplify a 76-bp DNA fragment corresponding to the Nc5 sequence (25). PCR reaction mixture (20 μl) contained: 10 μl of “FastStart Universal SYBR Green Master” (Rox) mix (Roche, United States); 0.8 μl of 20 μM of each primer (Genbiotech, Argentina); 7.4 μl of water, and 1 μl sample DNA. Parasite burden was expressed as the number of parasites/mg of bovine tissue. PCR positive samples were adjusted to a concentration of DNA of 20 ng/μL, and quantification of *N. caninum* DNA was performed by real-time PCR using the equipment ABI 7,500 FAST (Applied Biosystems, Foster City, CA, United States). The number of *N. caninum* tachyzoites was determined by interpolating the Ct values (cycle threshold value) on a standard curve. The standard curve was designed for the quantification of 10-1 to 10^5^ tachyzoites according to Regidor-Cerrillo et al. (26). To normalize the quantification of the parasite in each sample, a bovine β-actin standard curve was designed (from 64 ng of DNA per μL to 0.2 ng per μL). The results were expressed as the relation between parasite DNA and cell DNA amount (R2 ≥ 0.99; Slope values varied from −3.63 to −3.18).

### Genetic Characterization of *N. caninum* by Microsatellite Typing

Positive samples to conventional PCR were genetically characterized as described by Regidor-Cerrillo et al. (27). The aim of this typing was to genetically characterize circulating parasites and rule out the possibility of reinfection with a different *N. caninum* genotype. Briefly, 9 markers (MS4, MS5, MS6A, MS6B, MS7, MS8, MS10, MS12, and MS21) were amplified through multiplex nPCR (27). Peak Scanner TM v1.0 software (Applied Biosystems) to analyze the size of fluorescent PCR products. For automated allele sizing, fluorescent-labeled primers were used in the secondary PCR. Amplified products were prepared with HiDi formamide and Gene Scan-500 (LIZ) Size Standards (Applied Biosystems, CA, United States). The size of the fluorescent PCR product was determined using a 48-capillary 3,730 DNA analyzer (Applied Biosystems, CA, United States). The MS7 and MS10 sequences were analyzed using Big Dye Terminator v3.1 (Applied Biosystems) and a 3,730 DNA Analyzer (Applied Biosystems) at the Unidad Genómica del Parque Científico de Madrid.

### *In vitro N. caninum* Isolation

A total of 1,914 samples of PBMC obtained from animals belonging to the 4 experimental groups were assayed for *N. caninum* isolation. Cell culture was performed every 2 weeks from days 40 to 110 (*n* = 110) and weekly from days 110 to birth (*n* = 528).

The samples were washed 3 times with PBS at 1,000 *g* and seeded in triplicate at a concentration of 10^5^ cells/well in a 24 h VERO cell monolayer maintained with MEM culture, 1,000 IU/ml penicillin, and 100 mg/ml streptomycin supplemented with 2% fetal bovine serum, in 96 well-plates (Nunc®, New York, United States). After incubation for 24 h at 37°C, the cell culture medium was replaced. The plates were incubated at 37°C with 5% CO_2_. Cell cultures were daily examined with an inverted microscope (Olympus, Japan) and cultures were kept for 60 days (9).

### Congenital Transmission

The transplacental transmission was evaluated on precolostral serum taken from neonates using an indirect commercial ELISA (CIVTEST® BOVIS *Neospora*, HIPRA, Girona, Spain). Gamma-glutamyl transferase (GGT) activity was measured to assure the absence of colostrum intake before sampling using the γ-G-test according to manufacturer's instructions (Wiener Lab, Rosario, Argentina). GGT values above 50 IU/l are considered indicative of colostrum intake (28).

### Histopathological Studies on Placentas

Placental samples were taken after delivery. Several placentomes and the inter-cotyledonary chorion were fixed in 10% buffered formalin, processed by standard methods, and embedded in paraffin-wax blocks (29). About 4 μm-thick sections of tissue were cut, mounted on glass microscope slides, and stained with hematoxylin and eosin (H&E) and examined by microscopy (Olympus, Japan).

Tissues with *N. caninum*-compatible lesions were selected and analyzed by immunohistochemistry (IHC) as previously described by Campero et al. (29) with minor modifications. An Avidin-Biotin commercial kit (ABC Elite ABC Peroxidase Complex Vector PK6101, Vector, Burlingame, United States) was employed according to the instructions of the manufacturer. A rabbit polyclonal antibody against NC-1 tachyzoites was used as the primary antibody at 1: 300 dilution for an incubation period of 45 min at 37°C (kindly provided by Dr. Mark Anderson, UCDavis, Davis, United States). Secondary antibody (provided by the kit) was incubated for 30 min at 37°C and AEC substrate chromogen K3464 (Dako, Carpinteria, CA, United States) was incubated for 5 min at room temperature. Finally, sections were counterstained for 5 min with Mayer hematoxylin and rinsed with distilled water.

### Statistical Analysis

The chi-square test was used to evaluate *Neospora*-DNA detection in PBMC and the correlation between parasitemia, IFAT, IgG iELISA, IgG avidity ELISA. and IgG1/IgG2 iELISA was calculated by Pearson's correlation coefficient. Generalized linear models (GLM) based on Poisson distribution were used to compare total leukocytes, monocytes, neutrophils, eosinophils, and lymphocytes. GLM based on normal distribution was used for avidity analysis and Gamma distribution for IFN-γ, TNF-α, IL-10, and IL-12 analysis. SAS software (1987) was used and a *p* <0.05 was considered statistically significant. Graphics were performed with GraphPad Prism 5 v.5.01 software (San Diego, CA, United States).

## Results

### Calving

All gestating cows (+Preg and -−Preg groups) gave birth to clinically healthy calves. Body condition averaged between 3–4 (scale from 1 to 5) for all cows along with the study. One cow (#4) from +Preg group had placenta retention.

### Specific Humoral Immune Response in Cows and Neonates

**Figure 2:**
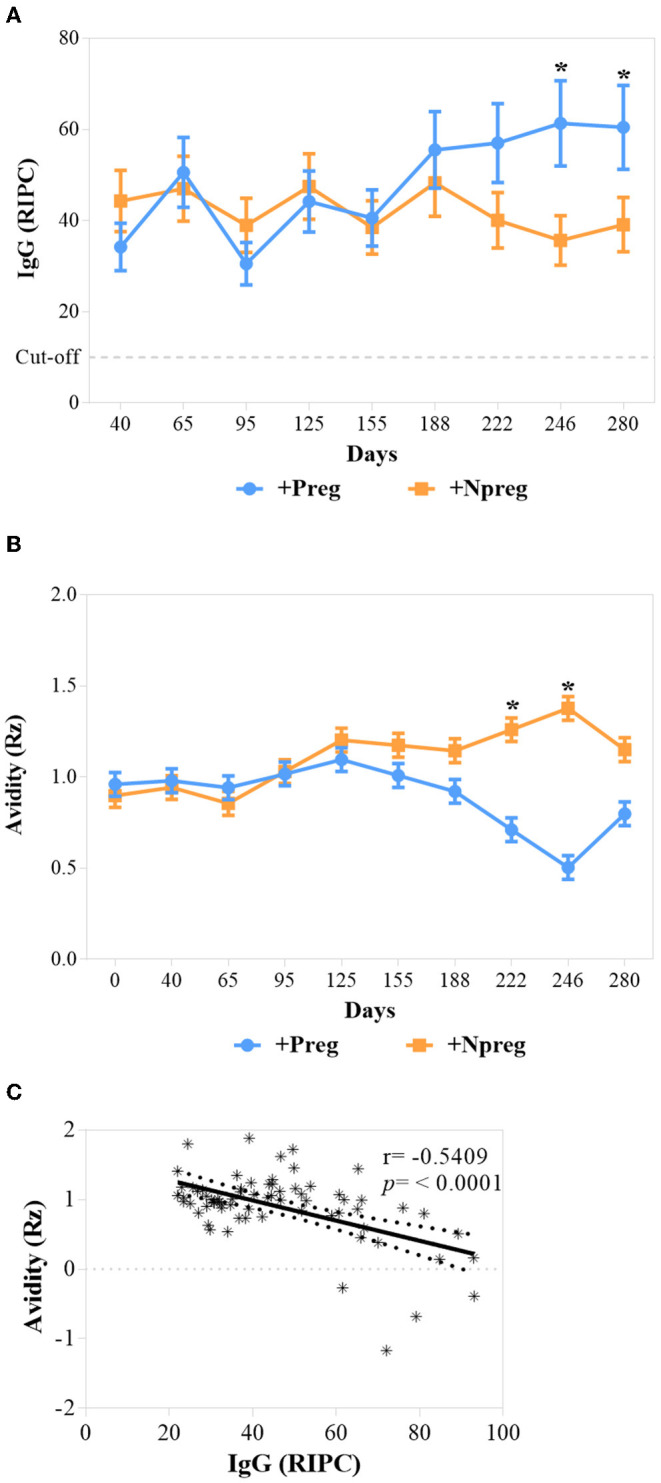
The concentration of IgG, cutoff: ≥10 RIPC **(A)**, and Avidity values (Rz) **(B)** of IgG from sera of *Neospora caninum* infected cows. Each point represents the mean ± standard error of the mean (SEM) at the different sampling times for groups +Preg and +Npreg. Correlations between Avidity (Rz) and RIPC for +Preg group **(C)**. The significant statistical differences between groups were analyzed: *p < 0.05.

Animals from all groups maintained the serological condition throughout the study according to the IFAT and ELISA results. Cows from +Preg group had the highest IgG anti-*N. caninum* concentration on days 246 and 280 compared to +Npreg group (*p* <0.0001) (Figure 2A).

Avidity was expressed as Rz value, where Rz ≤ 1 indicates high avidity, 1–2 intermediate, and ≥2 low avidity. Therefore, high avidity was detected on days 222 and 246 in the +Preg group (*p* <0.0281) (Figure 2B). A significant correlation (*r* = −0.5409; *p* <0.0001) was found between avidity (Rz value) and IgG (RIPC) in the +Preg group, thus indicating higher avidity at higher titers (Figure 2C). In addition, no correlation was found between these two variables in +Npreg group (*r* = −0.06363; *p* = 0.6008).

**Figure 3:**
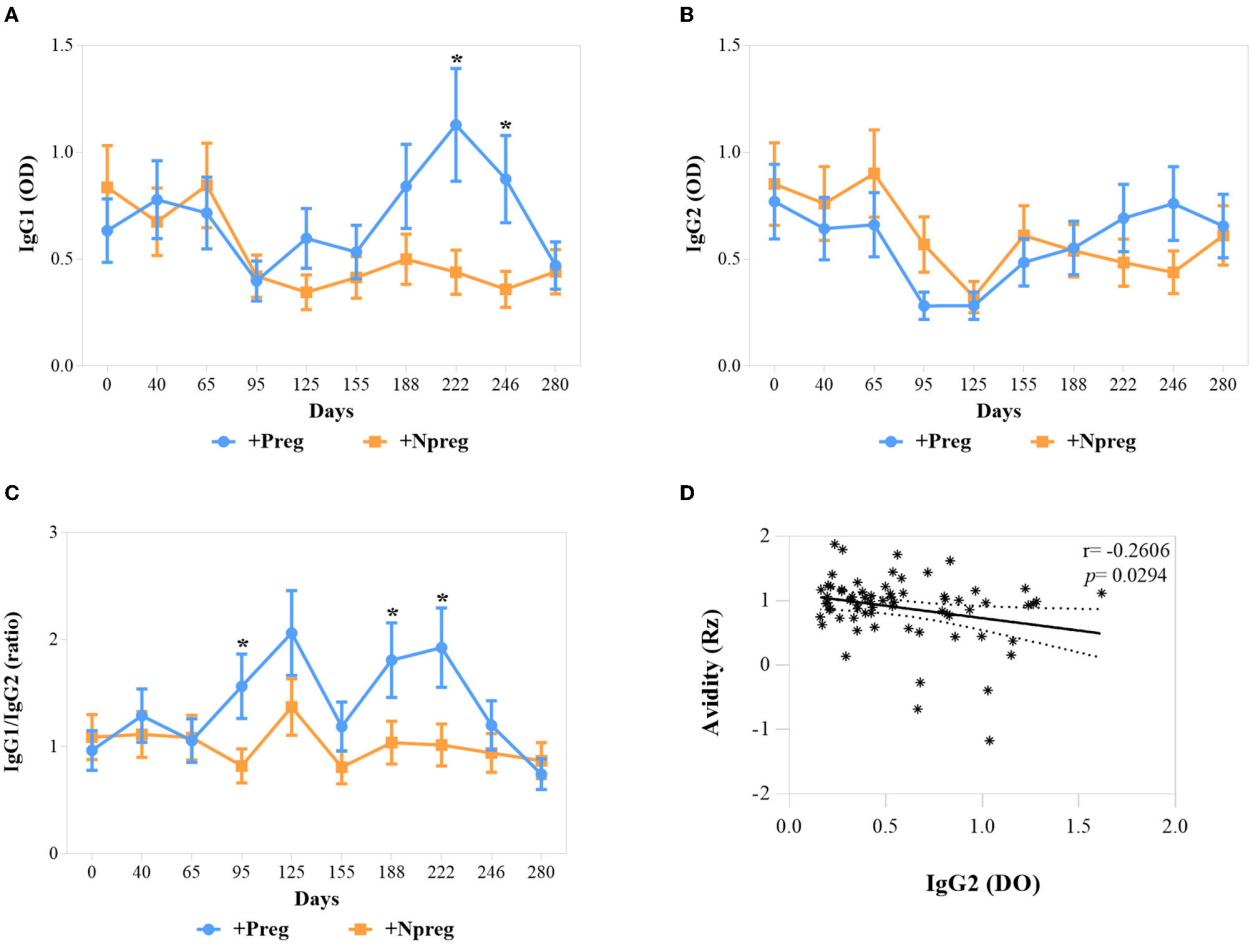
Optical densities (OD) of IgG1 **(A)**, IgG2 **(B)**, and IgG1/IgG2 ratio **(C)** in *Neospora caninum* seropositive cows. Each point represents the mean ± standard error of the mean (SEM) at the different sampling times for groups +Preg and +Npreg. Correlation between Avidity (Rz) and IgG2 **(D)**. The significant statistical differences between groups were analyzed: *p < 0.05.

IgG1 anti-*N. caninum* titers were significantly higher in the +Preg group on days 222 and 246 of the assay (*p* <0.0001) (Figure 3A). No significant differences in IgG2 values were detected between the 2 seropositive groups (Figure 3B). Significant differences in IgG1/IgG2 ratio between groups were detected on days 95, 188, and 222 of the assay (*p* <0.0174). In addition, the ratio increased in the second trimester and decreased in the last trimester of gestation in the +Preg group (Figure 3C). A significant correlation was detected between the Rz value and IgG2 OD (*r* = −0.2606; *p* = 0.0294) in the +Preg group, thus indicating higher avidity at higher titers of IgG2 (Figure 3D).

Colostrum samples from the +Preg group were positive for IgG anti-*N. caninum* with a mean RIPC of 63.85 (95% CI, 24.04–85.86). Colostrum samples from—the Preg group were negative and had a mean RIPC of 1.90 (95% CI, 0.59–3.07). The predominant IgG subclass in colostrum was IgG1.

Table 1 summarizes results on IgG subclasses, and avidity in neonates. Briefly, IgG1 was predominant in 86% of the neonates born from the +Preg group (6/7). Avidity of IgG anti- *N. caninum* varied with the following results: 3 neonates with low avidity values, 3 had intermediate, and 1 neonate had high avidity value. Neonates born from −Preg group all tested negative for *N. caninum* antibody detection tests.

**Table 1:** Precolostral IgG1, IgG2, IgG1/IgG2 ratio and avidity values in neonates born from *Neospora caninum* +Preg group.

**Neonate**	**IgG1 (OD)**	**IgG2 (OD)**	**IgG1/IgG2 (ratio)**	**Avidity (Rz value)**	**Avidity interpretation**
1	0.807	0.719	1.12	4.75	Low
2	0.907	0.139	6.53	1.62	Intermediate
3	0.565	0.713	0.79	1.49	Intermediate
4	0.391	0.082	4.77	3.12	Low
5	0.611	0.046	13.28	2.63	Low
6	0.989	0.159	6.22	0.19	High
7	0.474	0.247	1.92	1.53	Intermediate

### Non-Specific Immune Response: Leukocyte Counting and Cytokine Expression

**Figure 4:**
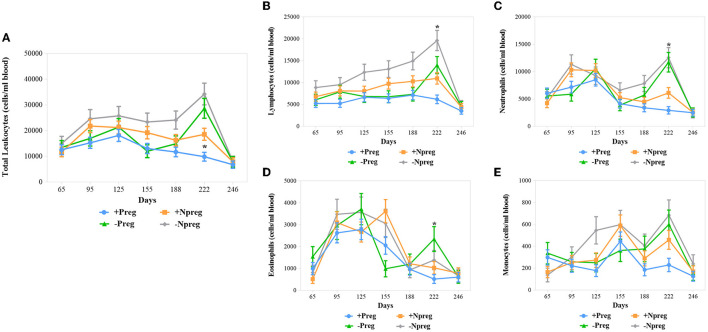
Mean leukocytes counting in blood from cows of four experimental groups. Total leukocytes **(A)** and absolute lymphocytes **(B)**; neutrophils **(C)**; eosinophils **(D)** and monocytes **(E)**. Each point represents the mean ± standard error of mean (SEM) at the different sampling times for groups. The significant statistical differences between groups were analyzed: *p < 0.05.

All 4 groups showed variation in the total leukocyte counting throughout the sampling although differences were only significant at 222 days where +Preg group had lower values compared to −Preg and −Npreg groups (*p* <0.0003) (Figure 4A). A drop of lymphocytes (Figure 4B), neutrophils (Figure 4C), and eosinophils (Figure 4D) was detected at 222 days of pregnancy in the +Preg group, with the lowest levels of lymphocytes and neutrophils (*p* <0.0001) compared to the other groups. A higher level of monocytes was observed in the −Npreg group compared to the +Preg group, mainly at 125 and 222 days, however, these differences were not significant (*p* > 0.05) (Figure 4E).

Cytokine expression in PBMC between groups was not significant at different sampling times analyzed (Supplementary Figure 1).

### *Neospora caninum* Molecular Detection

#### Frequency of Neospora-DNA in Blood in Cows and Neonates

**Figure 5:**
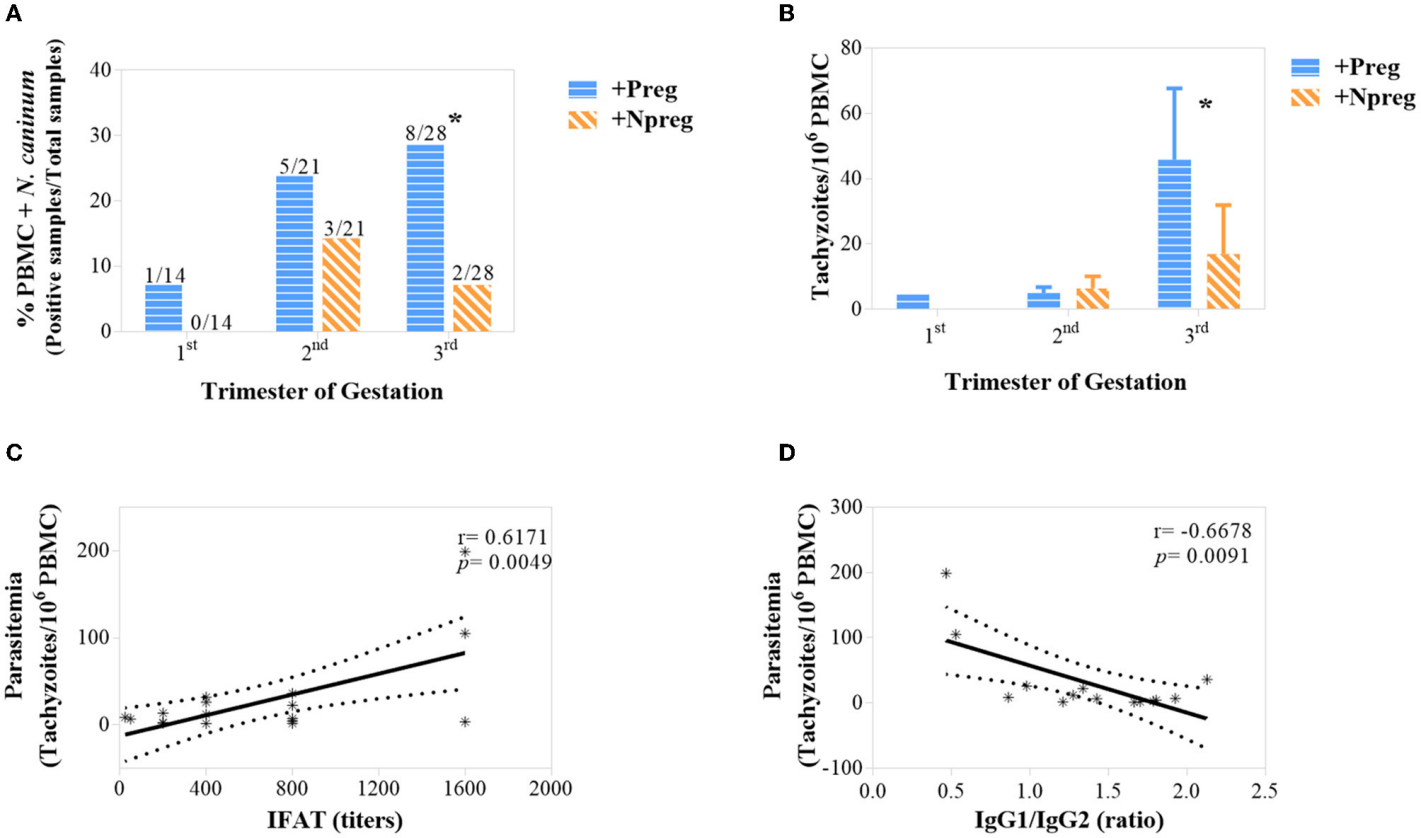
Percentage of *Neospora caninum* DNA detected in PBMC using conventional PCR along gestation. Bars represent the total of positive samples/total samples analyzed and *y*-axis represents % of positive samples **(A)**. *Neospora caninum* concentration (tachyzoites/10^6^ PBMC) along gestation. Bars represent the mean ± standard error of the mean (SEM) **(B)**. Correlations between parasitemia and IFAT **(C)** and IgG1/IgG2 ratio **(D)**. The significant statistical differences between groups were analyzed: *p < 0.05.

*Neospora caninum* DNA was detected in samples from +Preg and +Npreg groups in 6/7 and 3/7 cows, respectively (Supplementary Figure 2). Parasitemia was significant in the last trimester of gestation/trial where a higher frequency of *N. caninum* DNA was detected in blood in the +Preg group (Figure 5A) (*p* <0.00363). In addition, *Neospora*-DNA concentration was significantly higher in the +Preg group in the last third of gestation (Figure 5B) (*p* <0.05). A positive correlation was found between parasitemia and humoral response (IgG), being significant in relation to IFAT (*r* = 0.6171; *p* = 0.0049) (Figure 5C) but not in relation to iELISA (*r* = 0.3663; *p* = 0.1230). Interestingly, parasitemia also had a significant correlation with the IgG1/IgG2 ratio (*r* = −0.6678; *p* = 0.0091) in the +Preg group (Figure 5D). All blood samples from seronegative cows from −Preg and −Npreg groups were negative for PCR.

*Neospora caninum* DNA equivalent to 25.32 and 4.12 tachyzoites/10^6^ PBMC, was detected in 2/7 (29%) samples obtained from neonates born from the +Preg group, before colostrum intake. This frequency was similar to that detected in dams during the last trimester (*p* = 0.66). *Neospora caninum* DNA concentration also was similar to that observed in cows from +Preg and +NPreg groups. Neonates born from –the Preg group all tested negative for PCR.

#### Frequency of *N. caninum* DNA in Placentas

*Neospora caninum* DNA was detected on 6/7 (86%) placentas analyzed from the +Preg group. There was only one placenta that resulted negative to PCR from this group which had suffered autolysis due to placental retention. All placentas from -−Preg group resulted negative to PCR.

### Genetic Characterization of *N. caninum* in Cows and Neonates

Genetic characterization of *N. caninum* DNA in PBMC from cows and calves before colostrum intake, colostrum, and placentas is detailed in Table 2. Only a complete genotype from a placenta from cow #2 and a partial genotype from cow #3 of the group +Preg, was achieved. Most of the samples hence failed for achieving a complete genotyping profile, likely due to reduced DNA amount. All common MS markers identified in different samples (placenta, PBMCs, or colostrum) obtained from the same animal, including the polymorphic MS10, showed to be the same allele.

**Table 2:** Genotypes of *Neospora caninum* from placenta, colostrum, and PBMC of 5 cows and PBMC of one neonate belonging to the positive pregnant group (+Preg).

**Sample (cow#, tissue, section)**	MS4^**a**^ GC-*(AT)X*-ACATTT-(AT)2-AC*	**MS5 CG-*(TA)X*-TGTA-GG**	**MS6A GC-*(TA)X*-AC**	**MS6B CC-*(AT)X*-GT**	**MS7^**b**^ AT-TA-*(TA)X*-GG**	**MS8 TGAC-*(AT)X*-GG**	**MS10^**c**^ AGT-*(ACT)X*-*(AGA)Y*-*(TGA)Z*-CAA**	**MS12 GC-*(GT)X*-GC**	**MS21 TG-(TACA)3-TACC-*(TACA)X*-TT**
1 placenta C					15				
2 placenta D	13	10	13	11	10	13	6.16.7	16	9
2 placenta E					10	13	6.16.7	16	
3 placenta A						14		16	9
3 placenta D	11						6.15.8	16	
3 placenta E	11				11		6.15.8	16	9
5 PBMC	12				9.1				10
5 neonate PBMC								16	
5 colostrum									10
6 placenta A				11			6.13.9	16	
6 placenta C			14	11		18	6.13.9		

MS length (bp). ^a^Allele polymorphisms for each microsatellite are expressed as the number of repeats (X and X. Y and Z for MS10) in microsatellite sequences according to fragment size analysis and sequencing (27). ^b^The second number indicates the presence of a SNP. ^c^MS10 is a complex microsatellite with three different repetitive trinucleotide motives. Number of repeats for each repetitive motif is indicated by X. Y and Z. ^*^Microsatellite sequences. Repeated motif sequences are indicated by italics in the microsatellite sequence.

### *In vitro N. caninum* Isolation

*Neospora caninum* growth was not detected in any of the 1,914 samples from PBMC seeded in cell culture.

### Congenital Transmission

Precolostral IgG anti- *N. caninum* antibodies were detected in all calves born from +Preg group, thus vertical transmission was 100% efficient for this group (Supplementary Table 1). In addition, GGT activity was <50 UI/l in all serum samples.

### Histopathology

No significant lesions were detected microscopically in the placentas. Placentas from the -−Preg group did not show any lesions whereas placentas from the +Preg group had a mild multifocal lymphoplasmacytic placentitis characterized by a few foci of mild lymphoplasmacytic infiltrate, necrosis, and calcification (some samples) in the cotyledons and a mild lymphocytic and neutrophilic (some samples) infiltrate in the intercotyledonary zone (Supplementary Figure 3). All samples were negative for IHC.

## Discussion

This study is the first to evaluate *N. caninum* parasitemia by serial sampling/animal throughout the entire gestation/10-month period in naturally infected pregnant and non-pregnant beef cattle. Previous studies reported frequencies of *N. caninum* detection ranging from 0.8 to 80% (6, 7, 11–14). In addition, there are some works that were unsuccessful in detecting *N. caninum* from blood from naturally infected pregnant (30) and non-pregnant cattle (31). Multiple factors like the time-lapse between infection/recrudescence and sampling moment, circulating parasite load, or the frequency of sampling the same animal, may influence the efficiency of *N. caninum* detection. In our work, we detected frequencies of parasitemia of 22 and 8% in +Preg and +Npreg groups, respectively.

It has been proposed that *N. caninum* travels within PBMC to reach the placenta and spread the infection to the fetus (6). Okeoma et al. (10) and Santana et al. (12) reported higher frequencies of *N. caninum* DNA in the second and last third of gestation, coincidental with an increase in the antibody levels (1). Our results agree with these findings. Interestingly, we detected a positive correlation between parasitemia and humoral immune response. We detected a higher *N. caninum* DNA concentration in PBMC at delivery time, similar to Okeoma et al. (10). The immunosuppression that occurs near delivery could facilitate the cyst reactivation, explaining the higher DNA concentration detected at this moment (32). Also, immunosuppression may lead to leukopenia, neutrophilia, and lymphopenia at gestation (33). Our results reflect leukopenia and lymphopenia in the +Preg group, mainly at the end of gestation, but not in the −Preg group, while neutrophilia was not observed. Lower peripheral neutrophils count in the middle of the gestation period may be a consequence of neutrophils recruitment toward the foci of infection in an attempt of the dam to control parasite recrudescence at the placenta level (34, 35). Thus, our histopathological studies revealed neutrophil infiltrate in one placenta from the +Preg group. Monocytes peaked at 155 days of gestation similarly to Serrano et al. (35). They attributed this rise to a possible recrudescence of a chronic *N. caninum* infection.

The higher antibody concentration detected in the +Preg group could be associated with reactivation from tissue cyst during the second and last third of gestation. This increase is often associated with parasitemia, proliferation of *N. caninum* in the placenta, and an increase in the number of tachyzoites traveling back to the dam, and/or may also respond to estrogen stimulation (30, 36). As a consequence, the congenital transmission of *N. caninum* is favored (1, 37). Our results confirm these observations since we evidenced a positive and significant correlation between parasitemia and humoral immune response. In addition, the high vertical transmission rate (100%) detected in this study also reinforces this assumption.

Cows that deliver congenitally infected but clinically normal calves usually register higher antibody titers from half of gestation and then drop off 2 months post-partum (30, 36–38). In our study, we observed an antibody rise from half of gestation and a slight decrease toward the end of gestation, possibly to achieve an increase in antibody concentration in colostrum/milk (39).

The absence of significant differences in the cytokine expression in PBMC between groups at different sampling times agrees with Almería et al. (34) and Rosbottom et al. (40), suggesting that a Th1 or Th2 profile would not be so well-defined in cattle. In addition, Rosbottom et al. (40) observed a similar increase in the RNAm expression of IFNγ, IL-4, IL-12, IL-10, and TNF-α at the placenta in naturally infected cows, evidencing that in this organ, the immune response was not polarized toward either Th1 or a Th2 response.

Diverse results were reported on the IgG1/IgG2 ratio. Several authors detected an IgG1/IgG2 ratio <1, mainly in the second half of gestation in the cows that congenitally transmitted the infection to offspring in natural infections and experimentally infected pregnant cows (30, 41–43). However, in the cows where vertical transmission was not achieved and in experimental infection in heifers, the ratio was >1 (38, 41, 44). Stenlund et al. (36) observed that aborted and not aborted cows had IgG1/IgG2 >1 between the 5 and 7th months of gestation, being higher in the first group. In this study, all cows from the +Preg group transmitted the infection to offspring and a high IgG1/IgG2 ratio was detected from 95 to 246 days of gestation, similar to Stenlund et al. (36) and Andrianarivo et al. (42). From day 246, the IgG1/IgG2 ratio was low, which agrees with the results obtained by Williams et al. (38). The high IgG1/IgG2 ratio found at mid-gestation could be related to the Th2 response that usually occurs during pregnancy (5), whereas the low (<1) IgG1/IgG2 ratio found in the last month of pregnancy could be related to the parasite reactivation.

IgG1 subclass conforms to approximately 50% of the IgG present in the blood and is predominant in colostrum and milk from ruminants, being at an even higher concentration than IgA (45, 46). Our results on the IgG subclasses predominant in colostrum agree with the literature. A recent study by Maldonado-Rivera et al. (44) also identified IgG1 as the predominant subclass in the colostrum from *N. caninum* experimentally infected heifers. In addition, in 86% of the neonates born from the +Preg group, the predominant IgG1 subclass was also detected. Similarly to our finding, it has been reported that in congenitally infected calves, IgG1 is predominant (43, 44).

The variability of IgG avidity detected in neonates would indicate the moment of exposition to *N. caninum* during the fetal period. It is considered that a low avidity is indicative of a recent infection of up to 60 days, an intermediate avidity is indicative of an infection of 60–180 days ago, whereas a high avidity is found in infections higher than 6 months (1). Thus, in our study, neonates with low antibody avidity could have been infected after day 220 of gestation, while neonates with intermediate avidity between days 100 and 220 and neonates with high avidity could have been infected before day 100 of gestation.

The vertical transmission rate was 100% for the +Preg group. All neonates born in this group were healthy but congenitally infected. This value is slightly higher than the previous reports (1), however, the low number of animals evaluated may have overestimated the vertical transmission rate.

It has been proposed that *N. caninum* parasite load in the placenta drops to undetectable levels for the IHC technique after birth (1). In our study, even though IHC was negative in all samples, *N. caninum* DNA was detected in 86% of placentas. Salehi et al. (47) detected *N. caninum* DNA in 71% of placentas from naturally infected cows, similar to our findings. Based on our results, recrudescence and vertical transmission probably took place during the second and the beginning of the third trimester, therefore at delivery, high concentrations of the parasite in the placenta should not be expected, diminishing IHC detection.

Placental infection did not result in relevant lesions that affected the normal growth of the fetus or neonate survival. The calcifications detected may be related to the normal physiology of placentation and not necessarily related to neosporosis. Cantón et al. (48, 49) described mild lesions in placentas, as in our study, associated with infections at mid-gestation. Serological, molecular, and histopathological results from our study would seem to indicate that parasitemia and placental infection occurred mainly at late gestation.

A complete *N. caninum* genotype profile characterized by multilocus microsatellite was not achieved in most of the cases due to the low DNA concentration in the positive samples. However, we conclude that in 4 of the 5 cows in which genotyping was possible, at least one microsatellite differed. Furthermore, no more than a single genotype was detected per animal. This result suggests a lack of reinfections and supports the concept that parasitemia would have been caused by tissue cysts' reactivation. Three of the 4 cows with different *N. caninum* genotype belonged to the same herd; therefore, genetic differences would not be attributed to the origin of the animals. In addition, 2 sequences obtained for MS10 (cow #2 and #6) revealed no identity with *N. caninum* cattle isolates obtained from Argentina (50, 51) nor sequences reported on *Nespora*-aborted fetus DNA (52). The other MS10 sequence (cow #3) has already been reported (52).

In this study, we quantified *N. caninum* DNA in PBMC from naturally infected non-pregnant (first report) and pregnant cows. Also, we evaluated parasitemia, specific *N. caninum* antibody fluctuation, and avidity, throughout the entire gestation of seropositive and pregnant and non-pregnant cattle. *Neospora caninum* DNA in blood from congenitally infected calves is reported for the first time. Overall, *N. caninum* parasitemia is frequent in seropositive beef cows during the last third of gestation. This correlates with higher antibody levels and a decrease in total leukocyte counting. The IgG1/IgG2 ratio in cows infected with *N. caninum* is high at mid-pregnancy but low in the last period of gestation. The predominance of IgG1 subclass and avidity in neonates varies according to the moment of infection at gestation. We confirm the *N. caninum* infection in seropositive cattle through the DNA detected in the placenta and PBMC. The differences in the genotypes found have no relationship with the origin of animals. The precise timing of the parasitemia may be used for diagnosis purposes and/or for design strategies to avoid vertical transmission. Further studies are needed to identify the immune molecular mechanisms that favor parasitemia during gestation in chronically infected cattle.

## Data Availability Statement

The original contributions presented in the study are included in the article/supplementary material, further inquiries can be directed to the corresponding author/s.

## Ethics Statement

The animal study was reviewed and approved by CICUAE (CICUAE#009/2015).

## Author Contributions

IG and DM conceived and performed the study. LC and IG wrote the article. IG, YH, JR-C, ML, and ST conducted laboratory testing. IG, LC, SE, and JR-C analyzed the data. CC, AO, LO-M, IE, and DM provided funding acquisition, access to animals, and laboratory oversight. All authors read and approved the manuscript.

## Funding

This study was partially funded by the grant BID-PICT 2019-047, BID-PICT 2018-0638, Argentinean Government. *Neospora caninum* genotyping and cytokine mRNA expression was performed at SALUVET, UCM, Spain.

## Conflict of Interest

The authors declare that the research was conducted in the absence of any commercial or financial relationships that could be construed as a potential conflict of interest.

## Publisher's Note

All claims expressed in this article are solely those of the authors and do not necessarily represent those of their affiliated organizations, or those of the publisher, the editors and the reviewers. Any product that may be evaluated in this article, or claim that may be made by its manufacturer, is not guaranteed or endorsed by the publisher.

## Supplementary Material

Supplementary Figure 1 Relative cytokine mRNA expression levels in PBMC for experimental groups: IFN-γ **(A)**, IL-12 **(B)**, TNF-α **(C)**, and IL-10 **(D)**.

Supplementary Figure 2 Neospora caninum concentration at different sampling moments for cows from +Preg **(A)** and +Npreg groups **(B)**.

Supplementary Figure 3 Lymphoplasmacytic infiltrate foci (circle) in placenta from cow #3 from +Preg group **(A)**. Calcification areas (circles) in placenta from cow #2 from +Preg group **(B)**. HE stain. 100X.
