# Supplementary figures and images for: Parasitemia and Associated Immune Response in Pregnant and Non-Pregnant Beef Cows Naturally Infected With Neospora caninum
Source: Front Vet Sci. 2022 Jun 14;9:905271. doi: 10.3389/fvets.2022.905271 (PMC9238358; doi:10.3389/fvets.2022.905271)

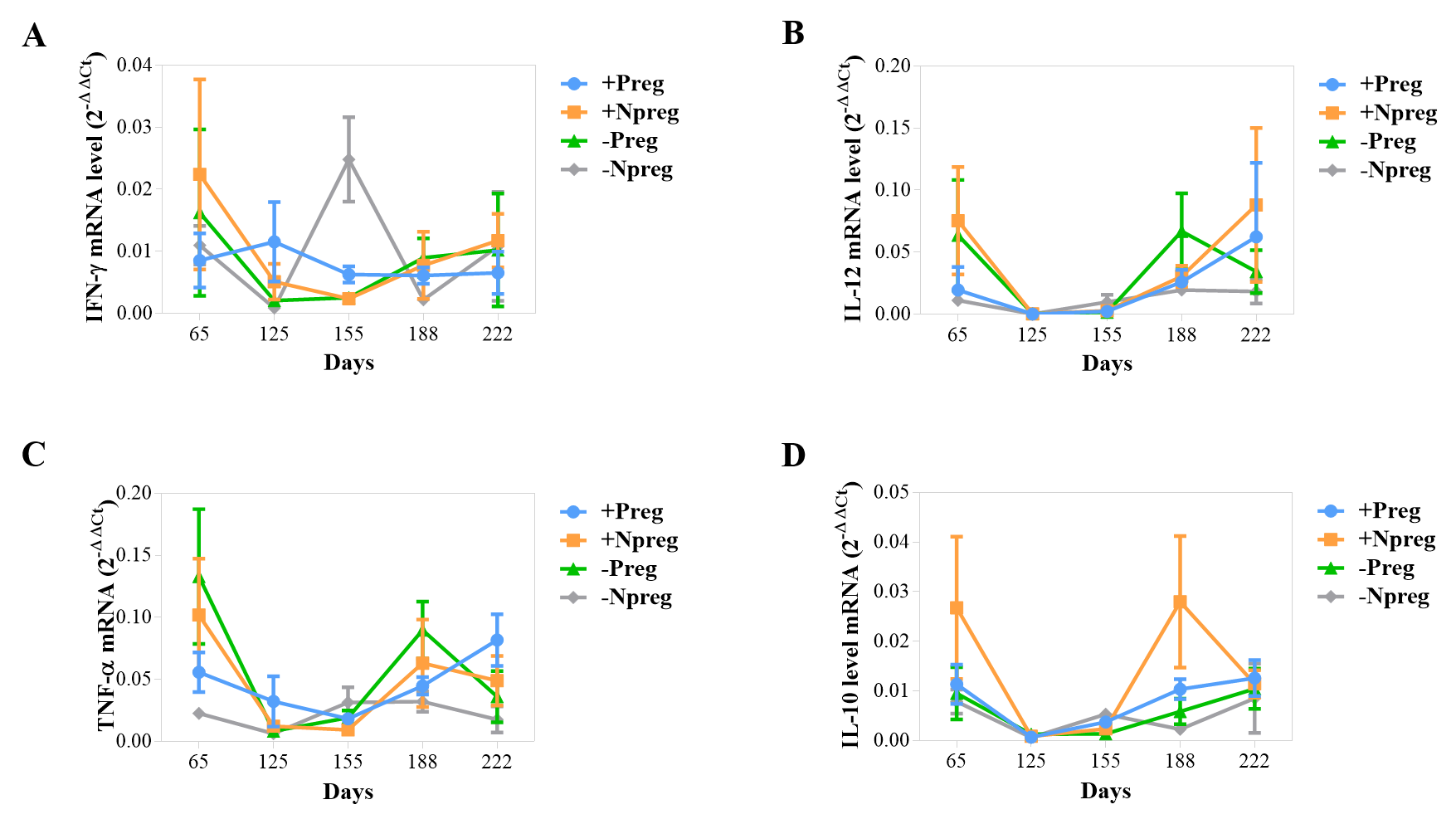

Supplement: Supplementary Figure 1 — Relative cytokine mRNA expression levels in PBMC for experimental groups: IFN-γ (A), IL-12 (B), TNF-α (C), and IL-10 (D). [file Image_1.TIF]

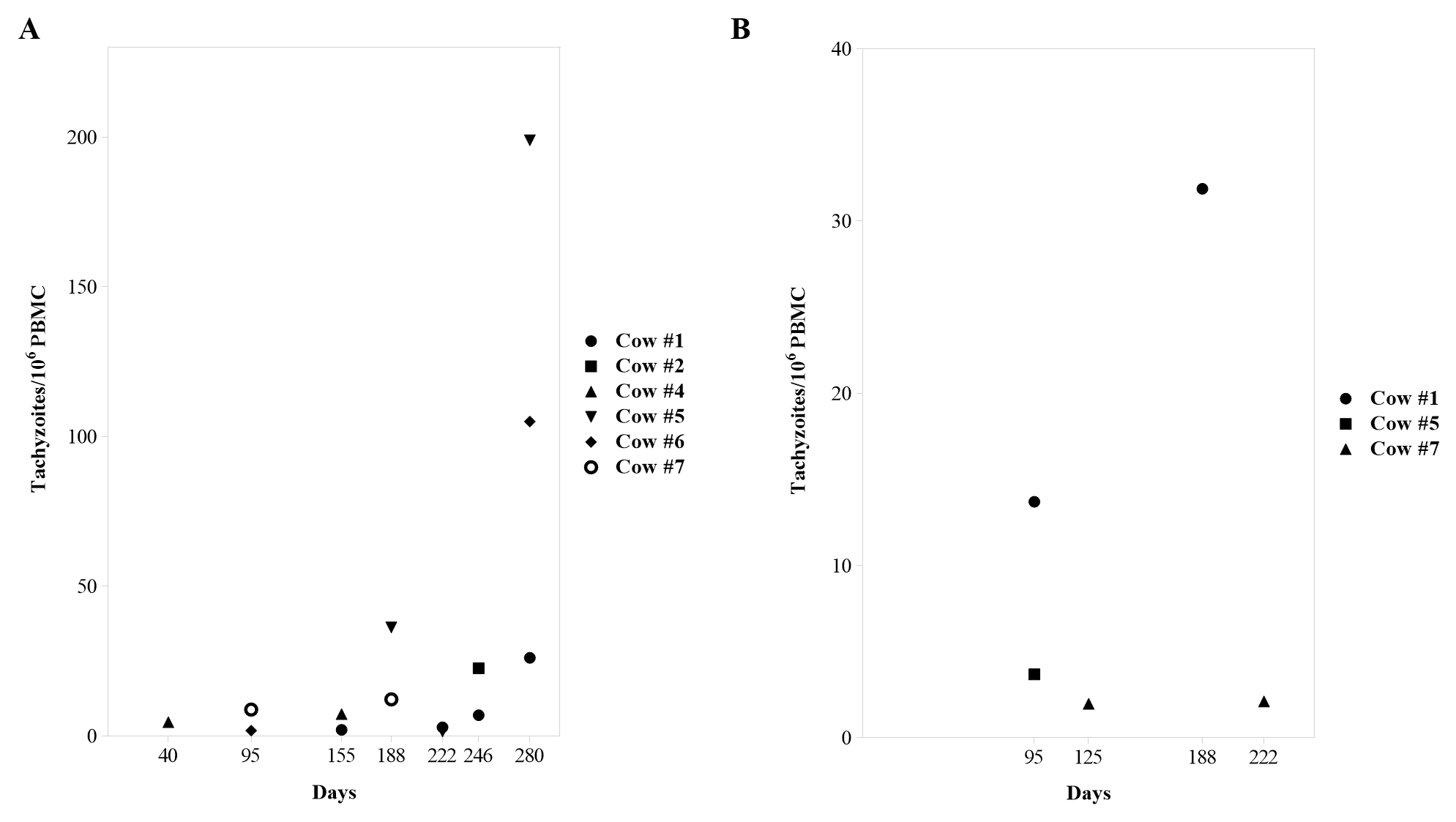

Supplement: Supplementary Figure 2 — Neospora caninum concentration at different sampling moments for cows from +Preg (A) and +Npreg groups (B). [file Image_2.TIF]

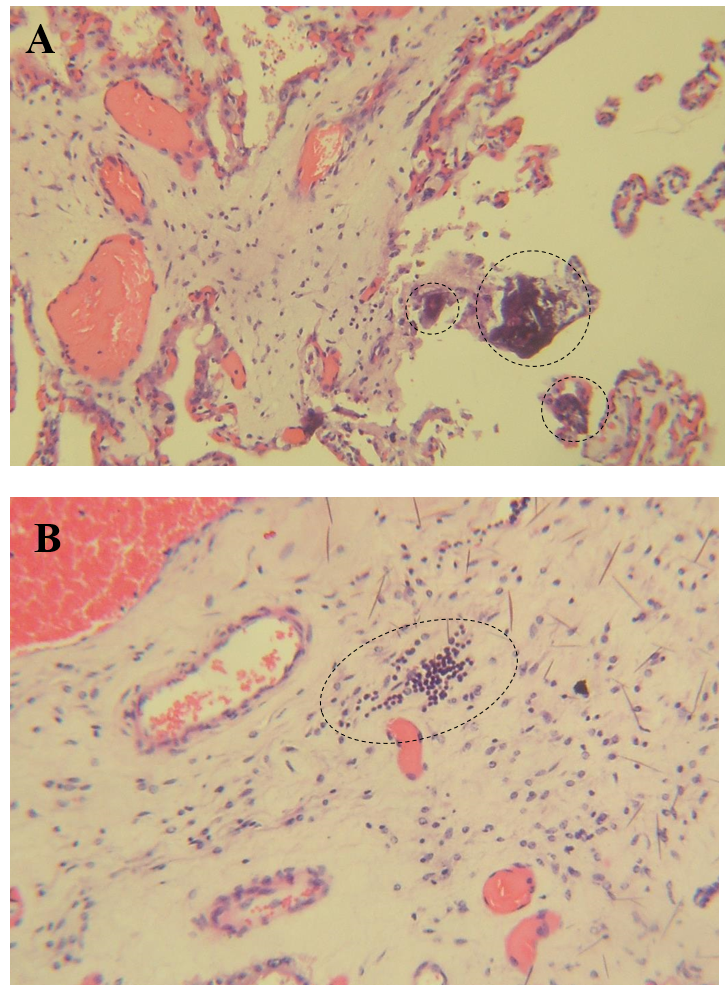

Supplement: Supplementary Figure 3 — Lymphoplasmacytic infiltrate foci (circle) in placenta from cow #3 from +Preg group (A). Calcification areas (circles) in placenta from cow #2 from +Preg group (B). HE stain. 100X. [file Image_3.tif]
