# Supplementary material for: Parasitemia and Associated Immune Response in Pregnant and Non-Pregnant Beef Cows Naturally Infected With Neospora caninum
Source: Front Vet Sci. 2022 Jun 14;9:905271. doi: 10.3389/fvets.2022.905271 (PMC9238358; doi:10.3389/fvets.2022.905271)
Supplement: Supplementary file 4 [file Table_1.docx]

**Table S1**. Precolostral IgG anti-*N. caninum* level and GGT activity in sera from calves born from –Preg and +Preg cows.

| **Group** | **Calves** | **RIPC** | **GGT (UI/l)** |
| --- | --- | --- | --- |
| -Preg | A | -5.29 | 2.3 |
|  | B | -4.48 | 2.3 |
|  | C | -5.45 | 2.7 |
|  | D | -3.35 | 4.6 |
| +Preg | 1 | 75.79 | 2.3 |
|  | 2 | 47.15 | 8.1 |
|  | 3 | 73.22 | 3.5 |
|  | 4 | 35.74 | 1.1 |
|  | 5 | 43.54 | 2.3 |
|  | 6 | 73.46 | 5.8 |
|  | 7 | 41.09 | 8.1 |
